# Improving Upper-Limb Recovery in Patients with Chronic Stroke Using an 8-Week Bilateral Arm-Training Device

**DOI:** 10.3390/life15070994

**Published:** 2025-06-22

**Authors:** Thanyaporn Wongwatcharanon, Pinailug Tantilipikorn Earde, Bunyong Rungroungdouyboon, Patcharee Kooncumchoo

**Affiliations:** 1 Medical Engineering Program, Faculty of Engineering, Thammasat University, Khlong Luang 12120, Thailand; thanyaporn.wongw@dome.tu.ac.th; 2 School of Human Kinetics and Health, Faculty of Health Science Technology, Chulabhorn Royal Academy, Bangkok 10210, Thailand; 3 Department of Physical Therapy, Faculty of Allied Health Sciences, Thammasat University, Khlong Luang 12120, Thailand; pinailug.e@allied.tu.ac.th; 4 Department of Mechanical Engineering, Faculty of Engineering, Thammasat University, Khlong Luang 12120, Thailand; rbunyong@engr.tu.ac.th; 5 Center of Excellence in Creative Engineering Design and Development, Thammasat University, Khlong Luang 12120, Thailand

**Keywords:** bilateral arm training, rehabilitation machine, stroke

## Abstract

Upper-limb impairments after stroke significantly affect patients’ quality of life and require effective rehabilitation strategies. Rehabilitation devices play a vital role in enhancing motor recovery. This study evaluated the efficacy of the *Arm Booster*, a bilateral arm-training device, in improving upper-limb impairment in patients with chronic stroke. Eighteen participants were randomly assigned to two groups: a device group (*n* = 9), using the *Arm Booster*; and a conventional physiotherapy group (*n* = 9). Both groups performed six bilateral upper-limb exercises (32 repetitions each) three times per week for eight weeks. Participants were further classified into mild spasticity (*n* = 5) and moderate-to-severe spasticity (*n* = 4) subgroups. The primary outcome was motor impairment, assessed using the Fugl-Meyer Assessment of the Upper Extremity (FMA-UE). Secondary outcomes included spasticity, measured by the Modified Ashworth Scale (MAS), and daily functional use of the arm, assessed with the Motor Activity Log (MAL). Both groups showed significant improvements in FMA-UE scores and overall arm movement. The conventional group demonstrated additional gains in hand and wrist function and coordination. Notably, in the moderate-to-severe spasticity subgroup, the device group exhibited improvements in upper-limb movement and a trend toward reduced spasticity. These findings suggest that the Arm Booster may support motor recovery, encourage the use of the affected arm, improve movement control, and provide an efficient means for patients to exercise more frequently on their own.

## 1. Introduction

Stroke is one of the most common conditions affecting the elderly and often results in widespread impairments, particularly in the upper and lower limbs. Upper-limb dysfunction—including weakness, impaired movement, poor motor control, and coordination deficits—significantly impacts daily living and self-care abilities [1], affecting up to 77% of stroke survivors [2]. These impairments not only limit basic self-care tasks but also negatively impact emotional and mental well-being [3,4], indicating an urgent need for effective rehabilitation strategies [5].

Rehabilitation aimed at improving upper-limb function is essential for maximizing patient outcomes and minimizing disability. A fundamental principle of effective rehabilitation is that training must occur with sufficient frequency and repetitions to promote motor control and motor learning. Generally, more frequent and intensive training is associated with better motor recovery. However, many patients face limitations that restrict access to consistent rehabilitation, leading to missed opportunities for recovery and suboptimal outcomes [6].

Bilateral arm training (BAT) has gained recognition as a theoretically sound and evidence-supported intervention in post-stroke upper-limb rehabilitation. This therapeutic strategy emphasizes synchronized bimanual activity, which facilitates engagement of the paretic limb and improves interlimb coordination through symmetrical motor execution [7]. The underlying theoretical mechanisms include interhemispheric rebalancing—wherein bilateral movement modulates transcallosal inhibition—and enhanced activation of ipsilesional motor cortices, both of which are believed to contribute to motor relearning and functional recovery [8]. Additionally, Sainburg et al. introduced the concept of “bilateral synergy” as a neurophysiological model, suggesting that cooperative control during bilateral movement minimizes maladaptive compensatory patterns while promoting more efficient cortical reorganization [9]. Critically, the effects of BAT are closely tied to neuroplasticity—the brain’s intrinsic capacity to reorganize neural pathways in response to practice and experience. Bilateral interventions engage both hemispheres simultaneously, promoting synaptic strengthening, the recruitment of residual or alternative neural circuits, and the restoration of interhemispheric balance. These neural adaptations are particularly important in stroke rehabilitation, where facilitating cortical remodeling is key to regaining motor function. Recent meta-analytical evidence supports this rationale, indicating that BAT significantly improves motor performance, especially in proximal limb segments, as assessed by clinical tools such as the Fugl-Meyer Assessment [10].

Recent advances in rehabilitation technology have significantly enhanced both the assessment and treatment of upper-limb dysfunction. Robotic and mechanical systems now enable high-precision, high-frequency, and repeatable movement training, supporting intensive and task-specific practice that facilitates neuroplastic changes and motor relearning [11,12]. These systems also support the development of individualized rehabilitation protocols that can be adapted in real time based on patient progress. A key element contributing to the success of these systems is the integration of real-time feedback mechanisms. Visual, auditory, and haptic feedback modalities serve to increase patient engagement, facilitate motor correction, and reinforce attention and sensorimotor control. Prior studies have demonstrated that feedback-based interventions lead to significant improvements in upper-limb function, especially when delivered in conjunction with repetitive, bilateral arm training, which promotes interhemispheric interaction and supports neuroplastic adaptation [13].

Importantly, embedding these technologies within the framework of the International Classification of Functioning, Disability and Health (ICF) allows for a more holistic and patient-centered approach to rehabilitation. The ICF framework not only addresses impairment reduction but also emphasizes activity participation, autonomy, and broader social determinants of recovery, such as financial well-being and long-term support in chronic stroke populations [14]. Collectively, these advancements highlight the critical need for the development of accessible and user-centered rehabilitation technologies that incorporate real-time feedback mechanisms, with the aim of optimizing both clinical effectiveness and functional outcomes in real-world settings for individuals undergoing post-stroke recovery.

This study aims to evaluate the effectiveness of a newly designed bilateral arm-training device, the Arm Booster, in comparison to conventional training for improving upper-limb function in chronic stroke patients. The *Arm Booster* allows patients to perform repetitive, high-frequency arm movements through the self-directed, simultaneous use of both arms. Designed to be user-friendly, simple, and accessible, the device seeks to expand rehabilitation opportunities. By offering an affordable option for self-guided therapy, the *Arm Booster* supports a return to functional independence and helps reduce the societal burden associated with stroke-related disabilities.

## 2. Materials and Methods

### 2.1. Participants

This controlled trial study was designed to evaluate the effectiveness of bilateral arm training in patients with chronic stroke over an 8-week period. Participants who met the eligibility criteria were recruited from the Medical and Rehabilitation Center and surrounding communities. The inclusion criteria were as follows: an age between 45 and 80 years; the first occurrence of stroke; a post-stroke duration of more than six months; a mild-to-moderate level of upper-limb impairment (FMA 20-66 points) [15,16]; the ability to sit independently for at least 30 min; the ability to understand and follow commands; and a stable medical condition.

The exclusion criteria included neurological disorders other than stroke; musculoskeletal conditions affecting upper-limb or hand movements [17]; severe arm spasticity, defined as a Modified Ashworth Scale (MAS) score greater than 3; a resting blood pressure exceeding 180/100 mmHg; Mini-Mental State Examination (MMSE) scores ≤14 for illiterate individuals, ≤17 for those with primary education, and ≤24 for those with secondary education; neglect or inattention to the contralateral side of the body; and current participation in rehabilitation or treatment targeting upper-limb or hand function.

Participants were randomly assigned to one of two groups: the device group, which received bilateral arm training with a novel machine; or the conventional group, which performed the same exercises without the device.

The sample size was calculated based on data from a previous study by Sethy et al. (2018) [18], using the Fugl-Meyer Assessment of the Upper Extremity (FMA-UE) and Motor Activity Log (MAL) as primary outcomes. A two-tailed test with 95% confidence (Z_α_ = 1.96) and 80% power (Z_β_ = 0.84) was assumed. Using means and standard deviations from the prior study (FMA-UE: μ_0_ = 44.78, μ_1_ = 36.92, σ = 3.22, d = 2.44; MAL: μ_0_ = 2.71, μ_1_ = 3.64, σ = 0.60, d = 1.55), the required sample size was estimated. Accounting for a 20% dropout rate, the final sample size was determined to be 18 participants (9 per group), based on the outcome measure (MAL) that yielded the highest sample size requirement [18].

### 2.2. Device Description

**Figure 1:**
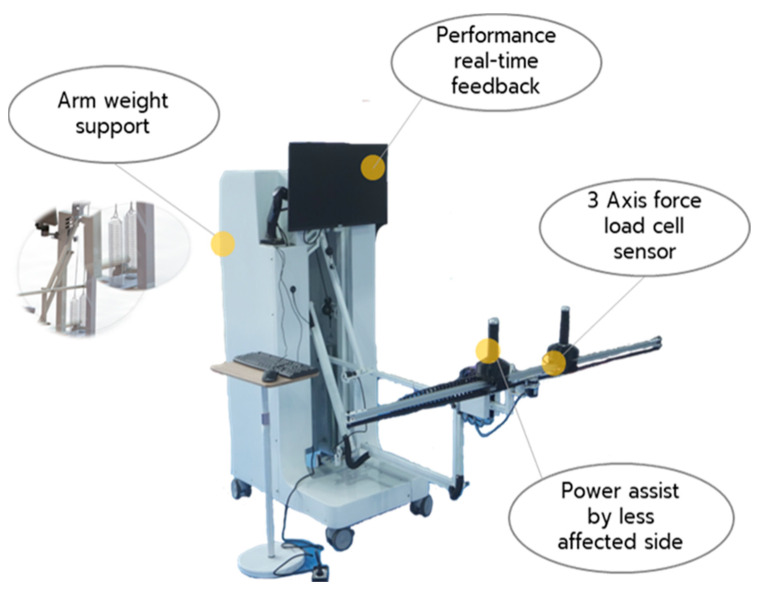
The components, highlighting the key features of the new bilateral arm-training device.

An overview of the key components and mechanical design of the *Arm Booster* is presented in Figure 1. Additional technical details can be found in a previous publication [19].

The bilateral arm-training machine, the *Arm Booster*, is a dual-arm device designed for upper-limb rehabilitation. It facilitates symmetrical movements by allowing the stronger arm to assist the weaker arm in performing coordinated actions. The device is equipped with sensors at both grip handles to measure the force exerted by each arm, enabling real-time comparison of force output. This feedback is valuable for both patients and physiotherapists, as it supports goal-oriented training by encouraging appropriate force generation and controlled movement.

The *Arm Booster* is a bilateral arm-training device developed to support upper-limb rehabilitation in individuals with chronic stroke. The system comprises three primary components: (1) a mechanical joint structure, (2) a sensor system, and (3) a processing and display system.

The mechanical joint structure consists of eight connecting rods affixed to both bases of the device, facilitating a predefined movement pattern that supports lifting and pressing motions of the upper limbs. The ends of the rods are connected to springs that serve to reduce the load on the grip handles by employing a counter-spring balance mechanism. This mechanical system incorporates slide rails and belts that allow force and motion transmission between the left and right grip handles, promoting symmetrical bimanual activity.

The sensor system is composed of force-detection sensors embedded within the grip handles. These sensors are designed to accurately quantify the force exerted by each arm during training. The real-time data provided by the sensors enable the monitoring of bilateral performance, offering both patients and therapists valuable feedback regarding force symmetry and engagement levels.

The processing and display system receives input from the force sensors, processes the data, and converts it into a percentage representation of exertion for each arm. These values are displayed in real time on an integrated screen, providing visual feedback that encourages active participation and increased effort during training sessions.

Collectively, these integrated systems enable the *Arm Booster* to deliver a structured and measurable rehabilitation experience. By combining mechanical assistance, sensor-based feedback, and real-time performance monitoring, the device promotes motor control, facilitates patient engagement, and assists therapists in tailoring interventions to individual patient needs.

### 2.3. Training Protocol

#### 2.3.1. Device Group

Participants in the intervention group received bilateral upper-limb training using the *Arm Booster*, a motion training device designed to facilitate symmetrical, high-repetition arm movements. The program comprised six standardized exercises targeting major upper-limb muscle groups: Horizontal shoulder abduction; elbow flexion; shoulder flexion; shoulder external rotation with elbow flexion; shoulder extension with elbow flexion; and shoulder flexion, abduction, and external rotation with supination. For each exercise, 32 repetitions were performed per session, with three sessions per week over a period of eight weeks.

#### 2.3.2. Control Group

Participants in the control group engaged in an exercise protocol identical to that of the intervention group in terms of movement type, session frequency, and overall duration. However, all training sessions were conducted without the use of assistive devices and were exclusively administered under therapist supervision. In this context, “therapist-guided” refers to individualized rehabilitation sessions provided by a licensed physiotherapist employing manual facilitation techniques.

Each session involved hands-on therapeutic approaches designed to promote accurate joint kinematics and correct maladaptive movement patterns. Throughout the intervention, the physiotherapist delivered real-time qualitative feedback—including corrective verbal instructions, postural adjustments, and, when necessary, physical assistance or resistance—to facilitate task-oriented motor activities.

### 2.4. Outcome Measures

Pre- and post-intervention assessments were conducted using standardized, validated tools to evaluate motor function, spasticity, and functional performance.

- Motor impairment and control were assessed using the Fugl-Meyer Assessment of the Upper Extremity (FMA-UE), which includes subdomains for arm movement, wrist movement, hand movement, coordination/speed, and a total score. The FMA-UE demonstrates excellent inter-rater reliability (r = 0.98–0.995) [20].
- Muscle spasticity was evaluated using the Modified Ashworth Scale (MAS), a clinical measure of resistance to passive movement. In this study, the MAS was applied specifically to the elbow flexor muscle group, with emphasis on the biceps brachii muscle, which is commonly affected in post-stroke upper-limb spasticity. Assessments were performed by a trained physiotherapist at the elbow joint, both at baseline and after the 8-week intervention. The MAS demonstrates excellent intra-rater reliability for elbow flexors (r = 0.84) [21]. To explore whether the spasticity level affected treatment outcomes, participants in the device group were further stratified into two subgroups based on the MAS score: mild spasticity: MAS < 1+ (*n* = 5), and moderate-to-severe spasticity: MAS ≤ 3 (*n* = 4).
- Functional performance in daily life was measured using the Motor Activity Log (MAL), which includes two components: the Amount of Use (AOU) and the Quality of Movement (QOM) scales. The AOU has high intra-rater reliability (r = 0.70–0.85), and the QOM shows acceptable test–retest reliability (r = 0.61–0.71) [22].

### 2.5. Statistical Analysis

The required sample size was calculated based on data from a previous study [18]. Data were analyzed using the SPSS software (version 22.0). The Kolmogorov–Smirnov test was applied to assess the normality of data distribution. Descriptive statistics were used to summarize participant characteristics and baseline data. Baseline comparisons were conducted using independent Student’s *t*-tests and chi-square tests. A two-way mixed-model ANOVA was used to analyze data for both within-group comparisons (pre- and post-intervention) and between-group or subgroup comparisons. Statistical significance was set at *p* < 0.05.

### 2.6. Ethical Considerations

The research protocol received ethical approval from the Human Research Ethics Committee of Thammasat University (Science) (HREC-TUSc; COA No. 082/2564), and informed consent was obtained from all participants. The study was registered with the Thai Clinical Trials Registry (TCTR) under the registration number TCTR20211130004.

## 3. Results

### 3.1. General Characteristic of Participants

A total of 18 participants completed the study and were evenly allocated to the device group (*n* = 9) and the conventional group (*n* = 9). No statistically significant differences were observed between the groups at baseline in terms of age, gender, stroke duration, or initial clinical scores. The detailed demographic and baseline characteristics are presented in Table 1.

### 3.2. Upper-Extremity Motor Impairment

Following the 8-week intervention, both groups demonstrated significant improvements in upper-limb motor function as measured by the Fugl-Meyer Assessment of Upper Extremity (FMA-UE). However, the specific domains of improvement differed between groups:

- In the device group, significant improvements were observed in arm movement, hand movement, and total FMA-UE score (*p* < 0.01).
- In the conventional group, significant gains were detected in arm movement (*p* < 0.05), wrist movement (*p* < 0.01), coordination/speed (*p* < 0.01), and the total score (*p* < 0.01).

When participants were stratified by spasticity level (Modified Ashworth Scale), the device group showed consistent improvements across both the mild and moderate-to-severe subgroups. In particular, the following was found:

- Participants with mild spasticity exhibited significant increases in arm movement and total FMA-UE score (*p* < 0.01).
- Those with moderate-to-severe spasticity also showed significant improvements in arm movement (*p* < 0.01) and total score (*p* < 0.05).

In contrast, the conventional group with moderate-to-severe spasticity showed no statistically significant changes in any FMA-UE subdomain. No significant between-group differences were found within either spasticity subgroup (Table 2).

### 3.3. Muscle Spasticity

Muscle tone, assessed by the Modified Ashworth Scale (MAS), revealed differential trends across groups:

- In the mild spasticity subgroup, 20% of participants in both groups demonstrated reductions in MAS scores post-intervention.
- In the moderate-to-severe subgroup, 50% of participants in the device group exhibited reduced spasticity, compared to 25% in the conventional group.

Although these observations suggest a potential advantage of device-assisted training for individuals with more pronounced spasticity, the between-group differences did not reach statistical significance.

### 3.4. Functional Performance

Functional use of the paretic arm, assessed using the Motor Activity Log (MAL), improved significantly in both groups:

- Among those with mild spasticity, both groups showed significant improvements on the Amount of Use (AOU) and Quality of Movement (QOM) subscales (*p* < 0.01).
- In the moderate-to-severe spasticity subgroup, only the device group demonstrated significant gains in both AOU and QOM (*p* < 0.01), while the conventional group showed improvement in AOU alone (*p* < 0.05), with no significant change in QOM.

No statistically significant differences in MAL scores were found between the two intervention groups post-assessment (Table 3).

## 4. Discussion

The present study examined the effects of an 8-week bilateral arm-training program using a bilateral arm-training machine in patients with chronic stroke. The findings demonstrated improvements in upper-extremity impairment and motor ability in both the device group and the conventional group. Although both groups showed positive outcomes, notable differences were observed between the two approaches, particularly in terms of motor function, spasticity reduction, and functional performance.

The improvements in motor function observed in this study are consistent with previous evidence suggesting that bilateral arm-training facilitates more effective recovery of proximal upper-limb function compared to unilateral training approaches [23]. This may be due to the concept of interhemispheric rebalancing achieved through bilateral symmetrical movements: increased activation in the lesioned hemisphere is associated with improved motor control in the affected arm [24]. Furthermore, the reduction in spasticity observed in this study is similar to previous findings, suggesting that structured rehabilitation programs—including passive and active assisted movements—can facilitate neuronal plasticity and reduce abnormal muscle tone [25].

Several studies have emphasized the importance of motor learning in post-stroke rehabilitation. Carr and Shepherd (1987) [26] highlighted the critical role of repetitive practice in acquiring motor skills, supporting the current findings that repeated symmetrical movements contribute to motor relearning and improved control. Furthermore, electromyography (EMG) studies have shown that bilateral movement training can facilitate more stable motor unit recruitment, resulting in smoother and more coordinated muscle activation patterns [27].

While these mechanisms provide plausible explanations for the observed improvements, it is important to note that the present study did not include direct neurophysiological assessments such as fNIRS, TMS, or EEG. Thus, the proposed mechanisms related to neuroplasticity and interhemispheric rebalancing remain theoretical and are inferred from the previous literature rather than directly measured outcomes. Future studies incorporating such neurophysiological measurements are warranted to validate and further elucidate the neural mechanisms underlying the benefits of bilateral arm training.

### 4.1. Effects on Spasticity and Muscle Control

A notable finding in this study was the differential response to training between patients with mild versus moderate-to-severe spasticity. In the mild spasticity subgroup, both training methods resulted in significant improvements in muscle coordination and function. This is consistent with previous studies demonstrating that active bilateral movement can reduce maladaptive co-activation patterns and enhance motor control [8].

In contrast, participants with moderate-to-severe spasticity appeared to benefit more from device-based training, which was associated with greater improvements in elbow extension and shoulder stability. These trends did not reach statistical significance and should be interpreted with caution. The findings are exploratory and warrant further investigation with adequately powered studies. These outcomes may be due to the mechanical assistance provided by the device, which likely helped to prevent undesired synergistic movements and enabled more isolated joint control.

The EMG analysis further supports this interpretation. Following the intervention, the device-trained group exhibited more refined EMG activity, characterized by smoother and more controlled activation patterns in the biceps brachii and infraspinatus muscles. These findings suggest improved voluntary muscle control and are consistent with the principle of motor unit synchronization, which posits that enhanced neuromuscular coordination improves movement efficiency [28].

### 4.2. Functional Implications

The improvements in functional performance, as assessed by the Motor Activity Log (MAL), suggest that enhanced muscle control translated into better real-world arm use. Participants in the device group demonstrated increased engagement of the affected arm in daily activities. This finding aligns with previous studies indicating that interventions involving symmetrical arm movements promote spontaneous use of the paretic limb [8]. Additionally, the improvement in hand function—supported by the cylindrical grip component of the training device—reinforces previous findings that task-specific grip exercises can strengthen neuronal pathways related to manual dexterity.

Interestingly, the impact of hand dominance on rehabilitation outcomes also warrants consideration. In the present study, participants with dominant-hand paresis exhibited greater improvements compared to those with non-dominant-hand involvement. This observation is consistent with Harris and Eng (2007) [29], who suggested that the dominant hand benefits from stronger neural representation, which may enhance recovery through more robust recruitment of residual motor pathways.

The *Arm Booster* device demonstrated notable efficacy in reducing upper-extremity impairments, particularly among participants with moderate-to-severe spasticity. While the conventional group showed statistically significant improvements in wrist motion and coordination/speed, these changes were not observed in the device group for the same measures. These improvements may reflect the benefits of therapist-guided adaptive training, which can be adjusted according to each participant’s abilities. However, the device group exhibited exclusive improvements in upper-extremity movement among those with higher spasticity levels. These findings suggest that the mechanical assistance and structured movement patterns provided by the device may offer greater benefit to individuals with more severe motor deficits.

Among participants with mild spasticity, the device group showed more pronounced post-training improvements in wrist motion and coordination/speed in the conventional group. This may reflect the benefit of physiotherapist-guided training, which incorporates hands-on techniques to guide correct movement patterns and reduce abnormal muscle tone. Physiotherapist-led sessions are designed to facilitate desired movements while inhibiting compensatory or maladaptive ones, thereby enhancing motor control and movement learning.

In contrast, the device group relied on self-directed movement control during repetitive tasks in various postural configurations. This active engagement likely contributed to neuroplastic changes through task-specific motor learning. The requirement for patients to control their movements independently may have promoted reorganization of motor pathways, supporting improvements in upper-limb and hand function. Notably, the device’s use of three-axis load cell sensors at the grip handles allowed for force feedback, encouraging precise and controlled movement. These features may have contributed to the observed improvements in movement quality and functional use of the affected limb.

### 4.3. Strengths, Limitations, and Future Directions

A major strength of this study is the comparison of two rehabilitation approaches in patients with chronic stroke with varying levels of spasticity. The inclusion of the EMG analysis provided objective evidence of neuromuscular changes, contributing to a deeper understanding of motor recovery mechanisms.

However, several limitations should be acknowledged. First, the device used in this study did not fully target distal-limb movements, potentially limiting its effect on fine motor control. Second, although real-time feedback was incorporated into the training protocol, future refinements of the device could include adaptive resistance features to enhance force modulation during movement. Third, the use of self-reported function scores, while clinically relevant, may introduce subjectivity in the assessment of motor improvements.

Another methodological limitation is the reliance on unadjusted mean differences without reporting effect sizes or using adjusted statistical techniques (e.g., ANCOVA or correction for multiple comparisons). This may limit the interpretability and generalizability of the findings, especially given the small sample size in the subgroup analyses. Future studies should incorporate these statistical methods to strengthen the reliability of the results.

Further research should investigate the long-term retention of training benefits and evaluate whether combining bilateral training with adjunctive therapies—such as functional electrical stimulation or virtual reality-based rehabilitation—could produce greater functional benefits. Additionally, individualized training protocols tailored to the severity of initial impairment should be explored to further optimize rehabilitation outcomes.

### 4.4. Clinical Significance

The analysis of total FMA-UE scores post-training among participants in the device group with mild spasticity revealed statistically significant improvements. When compared to the minimal clinically important difference (MCID) of 5.25 points, both the device and conventional groups exceeded this threshold, indicating clinically meaningful improvements in upper-limb function [30].

For participants with moderate-to-severe spasticity, the device appeared to reduce upper-extremity movement impairment. The device facilitates elbow extension through a mechanical lever system that includes power-assisted movement. This feature may help overcome reciprocal inhibition, a mechanism in which increased tone in the biceps brachii suppresses the activation of the extensor muscles. By promoting elbow extension and supporting sagittal plane movements—such as shoulder flexion—the device helps patients counteract gravitational forces, reduce abnormal co-contraction, and improve movement control.

Spasticity scores showed a reduction of 50% in the device group compared to 25% in the conventional group, suggesting a greater impact of device-assisted training on spasticity reduction. This may be due to the resistance generated by the biceps brachii during device-guided movements, which has been shown to influence spasticity positively [31]. Resistance training, that strengthens weakened muscles, is a well-recognized strategy in spasticity management [32].

Functional performance, as assessed by the Motor Activity Log (MAL), improved in both groups among participants with mild spasticity. These improvements may reflect reductions in motor impairment, leading to enhanced muscle coordination and control, which in turn support the better execution of functional tasks [33]. For participants with moderate-to-severe spasticity in the device training group, MAL scores also improved, indicating meaningful functional gains following the intervention.

Overall, the findings underscore the potential of the *Arm Booster* device to enhance rehabilitation outcomes for individuals with upper-limb impairments, particularly those with moderate-to-severe spasticity. The device’s ability to provide structured movement patterns and mechanical assistance appears especially beneficial for this subgroup, emphasizing the importance of individualized and targeted rehabilitation strategies.

Additionally, the observed improvements in wrist motion and coordination/speed within the device group suggest that integrating such technology into conventional rehabilitation programs may yield substantial benefits—particularly when combined with the clinical expertise of physiotherapists.

The clinical relevance of these improvements, as reflected by the minimal clinically important difference (MCID) scores, reinforces the device’s value in achieving meaningful functional gains. Moreover, the observed reduction in spasticity scores highlights the device’s potential role in managing muscle tone, which is critical for enhancing movement control and minimizing the impact of spasticity on daily activities.

## 5. Conclusions

This study provides preliminary evidence supporting the utility of the Arm Booster, a novel bilateral training device, in enhancing upper-limb motor outcomes in individuals with chronic stroke. Both device-assisted and conventional physiotherapy programs resulted in clinically meaningful improvements in motor function; however, the device group demonstrated additional advantages in managing spasticity and improving neuromuscular control, particularly in participants with moderate-to-severe impairment.

Importantly, the findings underscore the potential role of structured, self-directed, and high-repetition training in stroke rehabilitation. The use of sensor-based feedback and symmetrical movement patterns may offer unique benefits for promoting motor relearning and functional use of the affected limb. While these results are promising, the small sample size necessitates caution in generalizing the findings. Further large-scale, longitudinal studies are warranted to confirm these effects, explore long-term functional gains, and assess the integration of the device into multimodal rehabilitation strategies.

In summary, the Arm Booster may serve as a practical adjunct to traditional rehabilitation by facilitating patient engagement, supporting neuroplastic adaptation, and expanding access to task-specific training in individuals with chronic stroke.

## Figures and Tables

**Table 1:** Mean values, standard deviations, and percentages for general participant characteristics.

Characteristic	Conventional Group (*n* = 9) Mean ± SD	Device Group (*n* = 9) Mean ± SD	*p*-Value
Age (years)	65.33 ± 11.09	61.89 ± 5.62	0.418
Body mass index (kg/m^2^)	26.52 ± 4.91	27.48 ± 4.75	0.679
Duration of disease progression (years)	9 ± 5.81	6.34 ± 4.08	0.278
**Characteristic**	***n* (%)**	***n* (%)**	***p*** **-Value**
Gender			0.052
- Male	4 (44.44)	8 (88.89)	
- Female	5 (55.56)	1 (11.11)	
Type of stroke			1.000
- Ischemic stroke	9 (100)	9 (100)	
- Hemorrhagic stroke	-	-	
Weak side			0.372
- Left	5 (55.56)	3 (33.33)	
- Right	4 (44.44)	6 (66.67)	
Dominant arm			0.539
- Left	1 (11.11)	2 (22.22)	
- Right	8 (88.89)	7 (77.78)	
Modified Ashworth Scale (MAS) of the biceps brachii muscle			0.75
- Score of 1	3 (33.33)	3 (33.33)	
- Score of 1+	2 (22.22)	2 (22.22)	
- Score of 2	3 (33.33)	2 (22.22)	
- Score of 3	1 (11.11)	2 (22.22)	
Spasticity level			1.000
- Mild spasticity	5 (55.56)	5 (55.56)	
- Moderate-to-severe spasticity	4 (44.44)	4 (44.44)	

**Table 2:** Comparison of the impairment test results using the Fugl-Meyer Assessment of the Upper Extremity (FMA-UE) before and after the 8-week training period, comparing the group trained by physiotherapists (conventional group) and the group trained with the training device (device group).

Fugl-Meyer Assessment of the Upper Extremity (Scores)		Conventional Group			Device Group		
		Total (*n* = 9)	Mild Spasticity (*n* = 5)	Moderate-to-Severe Spasticity (*n* = 4)	Total (*n* = 9)	Mild Spasticity (*n* = 5)	Moderate-to-Severe Spasticity (*n* = 4)
Upper extremity (36)	Pre-test	24.11 ± 7.56	29.20 ± 5.93	17.75 ± 2.87	22.44 ± 5.175	26.00 ± 2.83	18.00 ± 3.65
	Post-test	25.56 ± 7.89 *	31.00 ± 5.96 *	18.75 ± 2.75	26.11 ± 6.03 **	30.60 ± 2.70 **	20.50 ± 3.42 **
	Mean difference	1.44 ± 0.53	1.80 ± 0.03	1.00 ± 0.61	3.67 ± 0.53	4.60 ± 0.0002	2.50 ± 0.61
Wrist (10)	Pre-test	3.78 ± 2.99	5.6 ± 2.51	1.50 ± 1.73	3.89 ± 2.67	5.8 ± 1.64	1.50 ± 1.29
	Post-test	4.44 ± 2.88 **	6.4 ± 2.07 *	2.00 ± 1.41	4.11 ± 2.67	6 ± 1.58	1.75 ± 1.50
	Mean difference	0.67 ± 0.20	0.80 ± 0.3	0.50 ± 0.27	0.22 ± 0.20	0.20 ± 0.3	0.25 ± 0.27
Hand (14)	Pre-test	7.00 ± 4.58	10.0 ± 3.39	3.25 ± 2.63	7.33 ± 2.12	8.6 ± 1.82	5.75 ± 1.26
	Post-test	7.44 ± 4.93	10.8 ± 3.42	3.25 ± 2.63	8.78 ± 3.03 **	11.0 ± 2.00 **	6.00 ± 0.82
	Mean difference	0.44 ± 0.44	0.80 ± 0.63	0.00 ± 0.18	1.44 ± 0.44	2.40 ± 0.63	0.25 ± 0.18
Coordination and speed (6)	Pre-test	1.89 ± 0.93	1.6 ± 0.55	2.25 ± 0.48	2.22 ± 0.44	2.2 ± 0.45	2.25 ± 0.48
	Post-test	2.78 ± 1.20 **	2.6 ± 1.14 *	3.00 ± 0.60	2.78 ±0.97	2.8 ± 1.10	2.75 ± 0.60
	Mean difference	0.89 ± 0.30	1.00 ± 0.42	0.75 ± 0.49	0.56 ± 0.30	0.60 ± 0.42	0.50 ± 0.49
Total (66)	Pre-test	36.78 ± 14.05	46.4 ± 11.04	24.75 ± 2.51	35.89 ± 9.49	42.6 ± 5.32	27.5 ± 2.51
	Post-test	40.22 ± 4.58 **	50.8 ± 9.98 **	27.00 ± 2.2	41.78 ± 1.13 **	50.4 ± 4.51 **	31.00 ± 2.2 *
	Mean difference	3.44 ± 0.92	4.40 ± 0.95	2.25 ± 1.19	5.89 ± 0.92	7.80 ± 0.95	3.50 ± 1.19

Notes: * indicates *p* < 0.05; ** indicates *p* < 0.01 compared to pre-training.

**Table 3:** A comparison of paretic arm usage in daily activities using the Motor Activity Log (MAL) before and after the 8-week training period, and between the conventional group and the device group.

Motor Activity Log (MAL)		Conventional Group		Device Group	
		Mild Spasticity (*n* = 5)	Moderate-to-Severe Spasticity (*n* = 4)	Mild Spasticity (*n* = 5)	Moderate-to-Severe Spasticity (*n* = 4)
Amount of Use [AOU]	Pre-test	13.50 ± 15.26	28.60 ± 14.17	37.20 ± 13.18	10.50 ± 6.14
	Post-test	16.75 ± 14.68 **	38.0 ± 6.17 *	48.2 ± 11.17 **	18.75 ± 4.57 **
	Mean difference	3.25 ± 2.16	9.40 ± 1.93	11.00 ± 1.93	8.25 ± 2.16
Quality of Movement [QOM]	Pre-test	14.00 ± 14.22	33.80 ± 21.25	45.20 ± 18.59	15.75 ± 12.55
	Post-test	16.75 ± 13.25 **	44.40 ± 20.42	56.40 ± 16.41 **	23.5 ± 11.82 **
	Mean difference	2.75 ± 2.19	10.60 ± 1.96	11.20 ± 1.96	7.75 ± 2.19

Notes: * indicates *p* < 0.05; ** indicates *p* < 0.01 compared to pre-training.

## Data Availability

The original contributions presented in the study are included in the article, further inquiries can be directed to the corresponding author.
